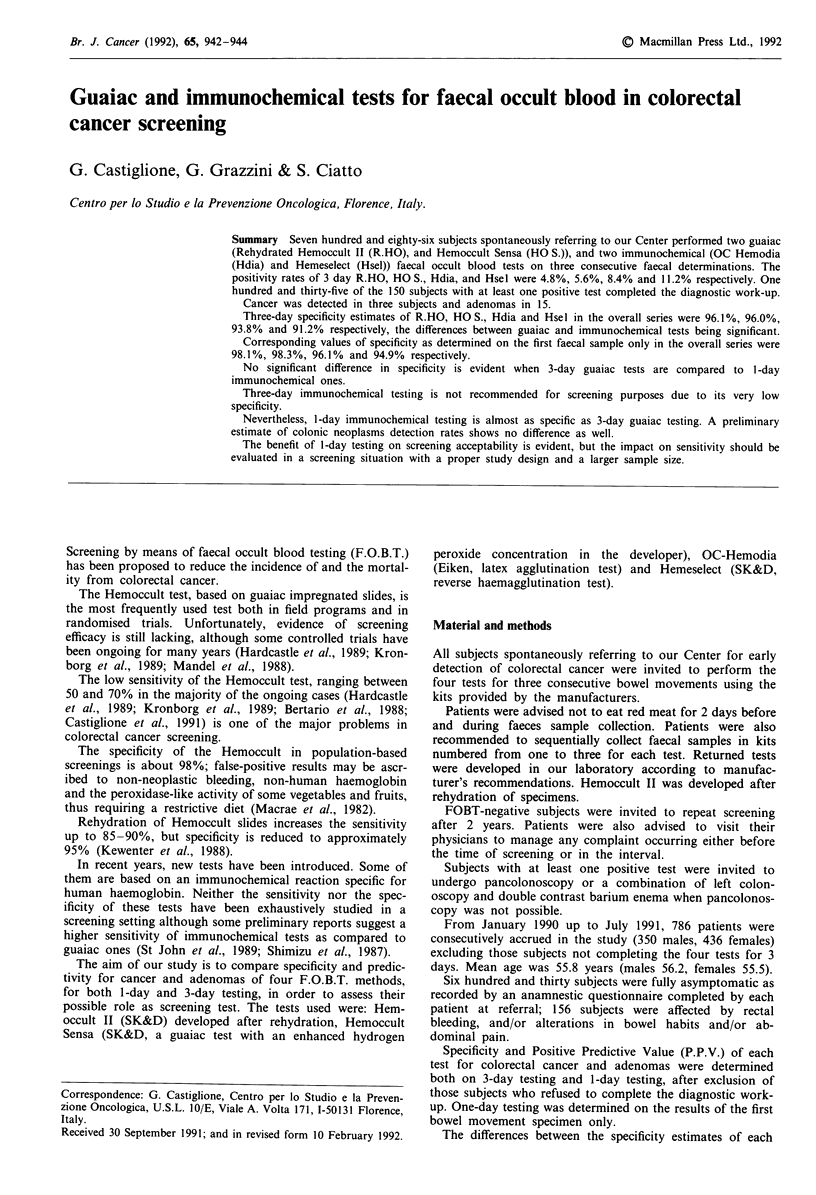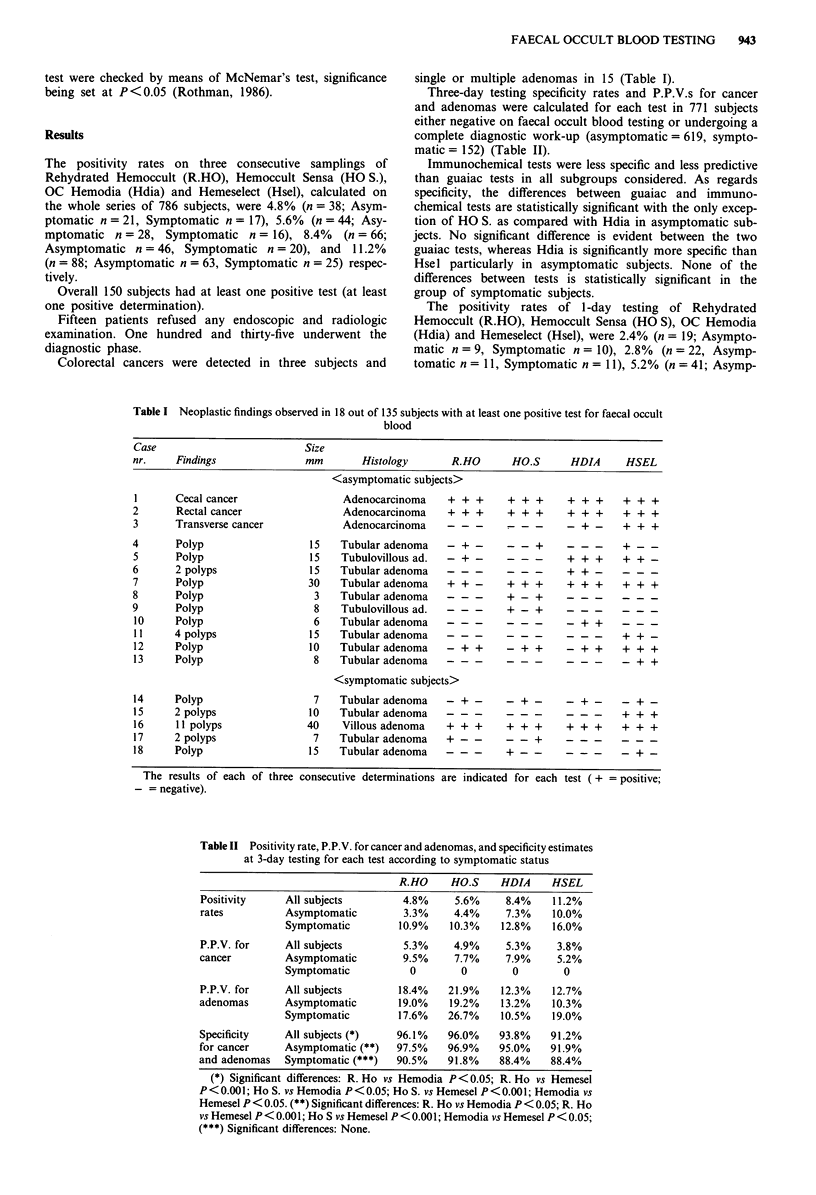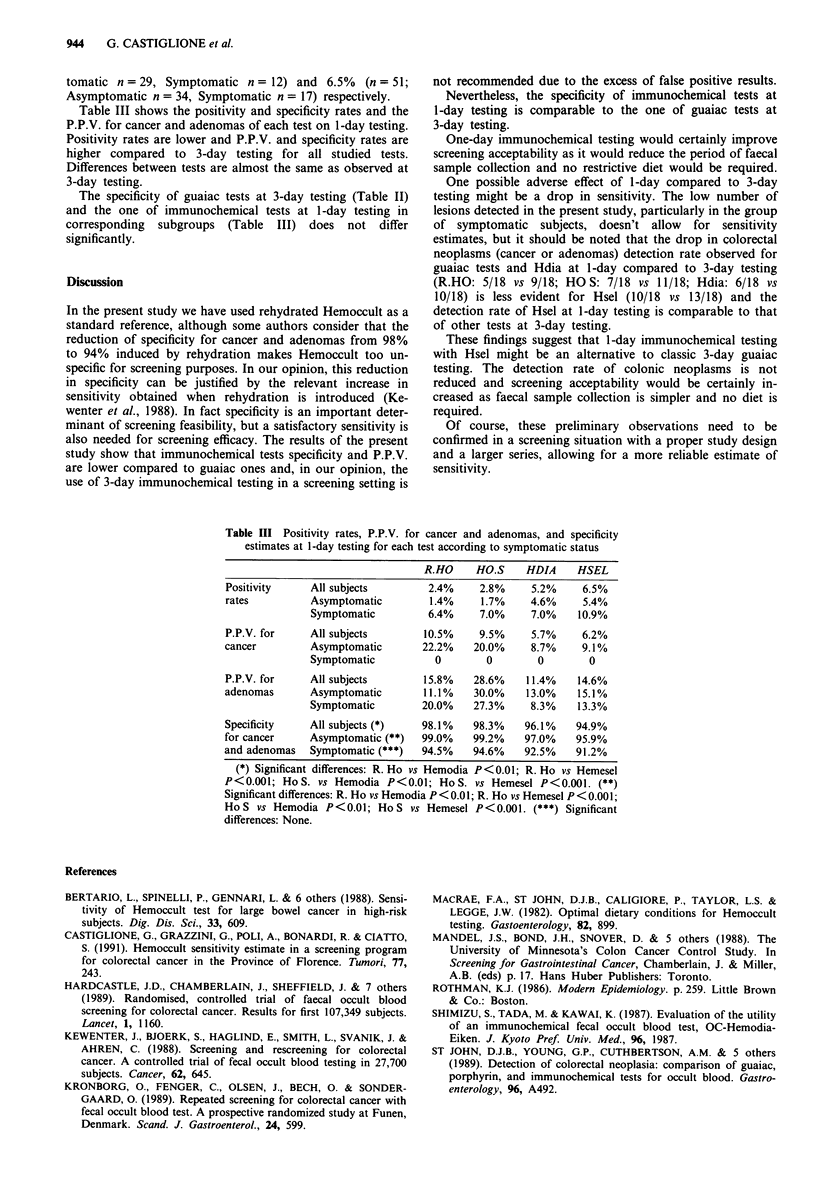# Guaiac and immunochemical tests for faecal occult blood in colorectal cancer screening.

**DOI:** 10.1038/bjc.1992.197

**Published:** 1992-06

**Authors:** G. Castiglione, G. Grazzini, S. Ciatto

**Affiliations:** Centro per lo Studio e la Prevenzione Oncologica, Florence, Italy.

## Abstract

Seven hundred and eighty-six subjects spontaneously referring to our Center performed two guaiac (Rehydrated Hemoccult II (R.HO), and Hemoccult Sensa (HO S.)), and two immunochemical (OC Hemodia (Hdia) and Hemeselect (Hsel)) faecal occult blood tests on three consecutive faecal determinations. The positivity rates of 3 day R.HO, HO S., Hdia, and Hsel were 4.8%, 5.6%, 8.4% and 11.2% respectively. One hundred and thirty-five of the 150 subjects with at least one positive test completed the diagnostic work-up. Cancer was detected in three subjects and adenomas in 15. Three-day specificity estimates of R.HO, HO S., Hdia and Hsel in the overall series were 96.1%, 96.0%, 93.8% and 91.2% respectively, the differences between guaiac and immunochemical tests being significant. Corresponding values of specificity as determined on the first faecal sample only in the overall series were 98.1%, 98.3%, 96.1% and 94.9% respectively. No significant difference in specificity is evident when 3-day guaiac tests are compared to 1-day immunochemical ones. Three-day immunochemical testing is not recommended for screening purposes due to its very low specificity. Nevertheless, 1-day immunochemical testing is almost as specific as 3-day guaiac testing. A preliminary estimate of colonic neoplasms detection rates shows no difference as well. The benefit of 1-day testing on screening acceptability is evident, but the impact on sensitivity should be evaluated in a screening situation with a proper study design and a larger sample size.


 © Macmillan Press Ltd., 1992

### Guaiac and immunochemical tests for faecal occult blood in colorectal cancer screening

G. Castiglione, G. Grazzini & S. Ciatto

*Centro per lo Studio e la Prevenzione Oncologica, Florence, Italy.*

**Summary** Seven hundred and eighty-six subjects spontaneously referring to our Center performed two guaiac (Rehydrated Hemoccult II (R.HO), and Hemoccult Sensa (HO S.)), and two immunochemical (OC Hemodia (Hdia) and Hemeselect (Hsel)) faecal occult blood tests on three consecutive faecal determinations. The positivity rates of 3 day R.HO, HO S., Hdia, and Hsel were 4.8%, 5.6%, 8.4% and 11.2% respectively. One hundred and thirty-five of the 150 subjects with at least one positive test completed the diagnostic work-up. Cancer was detected in three subjects and adenomas in 15.

Three-day specificity estimates of R.HO, HO S., Hdia and Hsel in the overall series were 96.1%, 96.0%, 93.8% and 91.2% respectively, the differences between guaiac and immunochemical tests being significant. Corresponding values of specificity as determined on the first faecal sample only in the overall series were 98.1%, 98.3%, 96.1% and 94.9% respectively.

No significant difference in specificity is evident when 3-day guaiac tests are compared to 1-day immunochemical ones.

Three-day immunochemical testing is not recommended for screening purposes due to its very low specificity.

Nevertheless, 1-day immunochemical testing is almost as specific as 3-day guaiac testing. A preliminary estimate of colonic neoplasms detection rates shows no difference as well.

The benefit of 1-day testing on screening acceptability is evident, but the impact on sensitivity should be evaluated in a screening situation with a proper study design and a larger sample size.

Screening by means of faecal occult blood testing (F.O.B.T.) has been proposed to reduce the incidence of and the mortality from colorectal cancer.

The Hemoccult test, based on guaiac impregnated slides, is the most frequently used test both in field programs and in randomised trials. Unfortunately, evidence of screening efficacy is still lacking, although some controlled trials have been ongoing for many years (Hardcastle *et al.*, 1989; Kronborg *et al.*, 1989; Mandel *et al.*, 1988).

The low sensitivity of the Hemoccult test, ranging between 50 and 70% in the majority of the ongoing cases (Hardcastle *et al.*, 1989; Kronborg *et al.*, 1989; Bertario *et al.*, 1988; Castiglione *et al.*, 1991) is one of the major problems in colorectal cancer screening.

The specificity of the Hemoccult in population-based screenings is about 98%; false-positive results may be ascribed to non-neoplastic bleeding, non-human haemoglobin and the peroxidase-like activity of some vegetables and fruits, thus requiring a restrictive diet (Macrae *et al.*, 1982).

Rehydration of Hemoccult slides increases the sensitivity up to 85-90%, but specificity is reduced to approximately 95% (Kewenter *et al.*, 1988).

In recent years, new tests have been introduced. Some of them are based on an immunochemical reaction specific for human haemoglobin. Neither the sensitivity nor the specificity of these tests have been exhaustively studied in a screening setting although some preliminary reports suggest a higher sensitivity of immunochemical tests as compared to guaiac ones (St John *et al.*, 1989; Shimizu *et al.*, 1987).

The aim of our study is to compare specificity and predictivity for cancer and adenomas of four F.O.B.T. methods, for both 1-day and 3-day testing, in order to assess their possible role as screening test. The tests used were: Hemoccult II (SK&D) developed after rehydration, Hemoccult Sensa (SK&D, a guaiac test with an enhanced hydrogen peroxide concentration in the developer), OC-Hemodia (Eiken, latex agglutination test) and Hemeselect (SK&D, reverse haemagglutination test).

Correspondence: G. Castiglione, Centro per lo Studio e la Prevenzione Oncologica, U.S.L. 10/E, Viale A. Volta 171, I-50131 Florence, Italy.

Received 30 September 1991; and in revised form 10 February 1992.

### Material and methods

All subjects spontaneously referring to our Center for early detection of colorectal cancer were invited to perform the four tests for three consecutive bowel movements using the kits provided by the manufacturers.

Patients were advised not to eat red meat for 2 days before and during faeces sample collection. Patients were also recommended to sequentially collect faecal samples in kits numbered from one to three for each test. Returned tests were developed in our laboratory according to manufacturer's recommendations. Hemoccult II was developed after rehydration of specimens.

FOBT-negative subjects were invited to repeat screening after 2 years. Patients were also advised to visit their physicians to manage any complaint occurring either before the time of screening or in the interval.

Subjects with at least one positive test were invited to undergo pancolonoscopy or a combination of left colonoscopy and double contrast barium enema when pancolonoscopy was not possible.

From January 1990 up to July 1991, 786 patients were consecutively accrued in the study (350 males, 436 females) excluding those subjects not completing the four tests for 3 days. Mean age was 55.8 years (males 56.2, females 55.5).

Six hundred and thirty subjects were fully asymptomatic as recorded by an anamnestic questionnaire completed by each patient at referral; 156 subjects were affected by rectal bleeding, and/or alterations in bowel habits and/or abdominal pain.

Specificity and Positive Predictive Value (P.P.V.) of each test for colorectal cancer and adenomas were determined both on 3-day testing and 1-day testing, after exclusion of those subjects who refused to complete the diagnostic work-up. One-day testing was determined on the results of the first bowel movement specimen only.

The differences between the specificity estimates of each test were checked by means of McNemar's test, significance being set at $P<0.05$ (Rothman, 1986).

### Results

The positivity rates on three consecutive samplings of Rehydrated Hemoccult (R.HO), Hemoccult Sensa (HO S.), OC Hemodia (Hdia) and Hemeselect (Hsel), calculated on the whole series of 786 subjects, were 4.8% ($n = 38$; Asymptomatic $n = 21$, Symptomatic $n = 17$), 5.6% ($n = 44$; Asymptomatic $n = 28$, Symptomatic $n = 16$), 8.4% ($n = 66$; Asymptomatic $n = 46$, Symptomatic $n = 20$), and 11.2% ($n = 88$; Asymptomatic $n = 63$, Symptomatic $n = 25$) respectively.

Overall 150 subjects had at least one positive test (at least one positive determination).

Fifteen patients refused any endoscopic and radiologic examination. One hundred and thirty-five underwent the diagnostic phase.

Colorectal cancers were detected in three subjects and single or multiple adenomas in 15 (Table I).

Three-day testing specificity rates and P.P.V.s for cancer and adenomas were calculated for each test in 771 subjects either negative on faecal occult blood testing or undergoing a complete diagnostic work-up (asymptomatic = 619, symptomatic = 152) (Table II).

Immunochemical tests were less specific and less predictive than guaiac tests in all subgroups considered. As regards specificity, the differences between guaiac and immunochemical tests are statistically significant with the only exception of HO S. as compared with Hdia in asymptomatic subjects. No significant difference is evident between the two guaiac tests, whereas Hdia is significantly more specific than Hsel particularly in asymptomatic subjects. None of the differences between tests is statistically significant in the group of symptomatic subjects.

The positivity rates of 1-day testing of Rehydrated Hemoccult (R.HO), Hemoccult Sensa (HO S), OC Hemodia (Hdia) and Hemeselect (Hsel), were 2.4% ($n = 19$; Asymptomatic $n = 9$, Symptomatic $n = 10$), 2.8% ($n = 22$, Asymptomatic $n = 11$, Symptomatic $n = 11$), 5.2% ($n = 41$; Asymp-

**Table I** Neoplastic findings observed in 18 out of 135 subjects with at least one positive test for faecal occult blood

| *Case nr.* | *Findings* | *Size mm* | *Histology* | *R.HO* | *HO.S* | *HDIA* | *HSEL* |
|---|---|---|---|---|---|---|---|
| | | | <asymptomatic subjects> | | | | |
| 1 | Cecal cancer | | Adenocarcinoma | + + + | + + + | + + + | + + + |
| 2 | Rectal cancer | | Adenocarcinoma | + + + | + + + | + + + | + + + |
| 3 | Transverse cancer | | Adenocarcinoma | − − − | − − − | − + − | + + + |
| 4 | Polyp | 15 | Tubular adenoma | − + − | − − + | − − − | + − − |
| 5 | Polyp | 15 | Tubulovillous ad. | − + − | − − − | + + + | + + − |
| 6 | 2 polyps | 15 | Tubular adenoma | − − − | − − − | + + − | − − − |
| 7 | Polyp | 30 | Tubular adenoma | + + − | + + + | + + + | + + + |
| 8 | Polyp | 3 | Tubular adenoma | − − − | + − + | − − − | − − − |
| 9 | Polyp | 8 | Tubulovillous ad. | − − − | + − + | − − − | − − − |
| 10 | Polyp | 6 | Tubular adenoma | − − − | − − − | − + + | − − − |
| 11 | 4 polyps | 15 | Tubular adenoma | − − − | − − − | − − − | + + − |
| 12 | Polyp | 10 | Tubular adenoma | − + + | − + + | − + + | + + + |
| 13 | Polyp | 8 | Tubular adenoma | − − − | − − − | − − − | − + + |
| | | | <symptomatic subjects> | | | | |
| 14 | Polyp | 7 | Tubular adenoma | − + − | − + − | − + − | − + − |
| 15 | 2 polyps | 10 | Tubular adenoma | − − − | − − − | − − − | + + + |
| 16 | 11 polyps | 40 | Villous adenoma | + + + | + + + | + + + | + + + |
| 17 | 2 polyps | 7 | Tubular adenoma | + − − | − − + | − − − | − − − |
| 18 | Polyp | 15 | Tubular adenoma | − − − | + − − | − − − | − + − |

The results of each of three consecutive determinations are indicated for each test ( + = positive; − = negative).

**Table II** Positivity rate, P.P.V. for cancer and adenomas, and specificity estimates at 3-day testing for each test according to symptomatic status

| | | *R.HO* | *HO.S* | *HDIA* | *HSEL* |
|---|---|---|---|---|---|
| Positivity rates | All subjects | 4.8% | 5.6% | 8.4% | 11.2% |
| | Asymptomatic | 3.3% | 4.4% | 7.3% | 10.0% |
| | Symptomatic | 10.9% | 10.3% | 12.8% | 16.0% |
| P.P.V. for cancer | All subjects | 5.3% | 4.9% | 5.3% | 3.8% |
| | Asymptomatic | 9.5% | 7.7% | 7.9% | 5.2% |
| | Symptomatic | 0 | 0 | 0 | 0 |
| P.P.V. for adenomas | All subjects | 18.4% | 21.9% | 12.3% | 12.7% |
| | Asymptomatic | 19.0% | 19.2% | 13.2% | 10.3% |
| | Symptomatic | 17.6% | 26.7% | 10.5% | 19.0% |
| Specificity for cancer and adenomas | All subjects (*) | 96.1% | 96.0% | 93.8% | 91.2% |
| | Asymptomatic (**) | 97.5% | 96.9% | 95.0% | 91.9% |
| | Symptomatic (***) | 90.5% | 91.8% | 88.4% | 88.4% |

(*) Significant differences: R. Ho *vs* Hemodia $P<0.05$; R. Ho *vs* Hemesel $P<0.001$; Ho S. *vs* Hemodia $P<0.05$; Ho S. *vs* Hemesel $P<0.001$; Hemodia *vs* Hemesel $P<0.05$. (**) Significant differences: R. Ho *vs* Hemodia $P<0.05$; R. Ho *vs* Hemesel $P<0.001$; Ho S *vs* Hemesel $P<0.001$; Hemodia *vs* Hemesel $P<0.05$; (***) Significant differences: None.

tomatic $n = 29$, Symptomatic $n = 12$) and 6.5% ($n = 51$; Asymptomatic $n = 34$, Symptomatic $n = 17$) respectively.

Table III shows the positivity and specificity rates and the P.P.V. for cancer and adenomas of each test on 1-day testing. Positivity rates are lower and P.P.V. and specificity rates are higher compared to 3-day testing for all studied tests. Differences between tests are almost the same as observed at 3-day testing.

The specificity of guaiac tests at 3-day testing (Table II) and the one of immunochemical tests at 1-day testing in corresponding subgroups (Table III) does not differ significantly.

### Discussion

In the present study we have used rehydrated Hemoccult as a standard reference, although some authors consider that the reduction of specificity for cancer and adenomas from 98% to 94% induced by rehydration makes Hemoccult too unspecific for screening purposes. In our opinion, this reduction in specificity can be justified by the relevant increase in sensitivity obtained when rehydration is introduced (Kewenter *et al.*, 1988). In fact specificity is an important determinant of screening feasibility, but a satisfactory sensitivity is also needed for screening efficacy. The results of the present study show that immunochemical tests specificity and P.P.V. are lower compared to guaiac ones and, in our opinion, the use of 3-day immunochemical testing in a screening setting is not recommended due to the excess of false positive results.

Nevertheless, the specificity of immunochemical tests at 1-day testing is comparable to the one of guaiac tests at 3-day testing.

One-day immunochemical testing would certainly improve screening acceptability as it would reduce the period of faecal sample collection and no restrictive diet would be required.

One possible adverse effect of 1-day compared to 3-day testing might be a drop in sensitivity. The low number of lesions detected in the present study, particularly in the group of symptomatic subjects, doesn't allow for sensitivity estimates, but it should be noted that the drop in colorectal neoplasms (cancer or adenomas) detection rate observed for guaiac tests and Hdia at 1-day compared to 3-day testing (R.HO: 5/18 *vs* 9/18; HO S: 7/18 *vs* 11/18; Hdia: 6/18 *vs* 10/18) is less evident for Hsel (10/18 *vs* 13/18) and the detection rate of Hsel at 1-day testing is comparable to that of other tests at 3-day testing.

These findings suggest that 1-day immunochemical testing with Hsel might be an alternative to classic 3-day guaiac testing. The detection rate of colonic neoplasms is not reduced and screening acceptability would be certainly increased as faecal sample collection is simpler and no diet is required.

Of course, these preliminary observations need to be confirmed in a screening situation with a proper study design and a larger series, allowing for a more reliable estimate of sensitivity.

**Table III** Positivity rates, P.P.V. for cancer and adenomas, and specificity estimates at 1-day testing for each test according to symptomatic status

| | | *R.HO* | *HO.S* | *HDIA* | *HSEL* |
|---|---|---|---|---|---|
| Positivity rates | All subjects | 2.4% | 2.8% | 5.2% | 6.5% |
| | Asymptomatic | 1.4% | 1.7% | 4.6% | 5.4% |
| | Symptomatic | 6.4% | 7.0% | 7.0% | 10.9% |
| P.P.V. for cancer | All subjects | 10.5% | 9.5% | 5.7% | 6.2% |
| | Asymptomatic | 22.2% | 20.0% | 8.7% | 9.1% |
| | Symptomatic | 0 | 0 | 0 | 0 |
| P.P.V. for adenomas | All subjects | 15.8% | 28.6% | 11.4% | 14.6% |
| | Asymptomatic | 11.1% | 30.0% | 13.0% | 15.1% |
| | Symptomatic | 20.0% | 27.3% | 8.3% | 13.3% |
| Specificity for cancer and adenomas | All subjects (*) | 98.1% | 98.3% | 96.1% | 94.9% |
| | Asymptomatic (**) | 99.0% | 99.2% | 97.0% | 95.9% |
| | Symptomatic (***) | 94.5% | 94.6% | 92.5% | 91.2% |

(*) Significant differences: R. Ho *vs* Hemodia $P < 0.01$; R. Ho *vs* Hemesel $P < 0.001$; Ho S. *vs* Hemodia $P < 0.01$; Ho S. *vs* Hemesel $P < 0.001$. (**) Significant differences: R. Ho *vs* Hemodia $P < 0.01$; R. Ho *vs* Hemesel $P < 0.001$; Ho S *vs* Hemodia $P < 0.01$; Ho S *vs* Hemesel $P < 0.001$. (***) Significant differences: None.

### References

BERTARIO, L., SPINELLI, P., GENNARI, L. & 6 others (1988). Sensitivity of Hemoccult test for large bowel cancer in high-risk subjects. *Dig. Dis. Sci.*, **33**, 609.

CASTIGLIONE, G., GRAZZINI, G., POLI, A., BONARDI, R. & CIATTO, S. (1991). Hemoccult sensitivity estimate in a screening program for colorectal cancer in the Province of Florence. *Tumori*, **77**, 243.

HARDCASTLE, J.D., CHAMBERLAIN, J., SHEFFIELD, J. & 7 others (1989). Randomised, controlled trial of faecal occult blood screening for colorectal cancer. Results for first 107,349 subjects. *Lancet*, **1**, 1160.

KEWENTER, J., BJOERK, S., HAGLIND, E., SMITH, L., SVANIK, J. & AHREN, C. (1988). Screening and rescreening for colorectal cancer. A controlled trial of fecal occult blood testing in 27,700 subjects. *Cancer*, **62**, 645.

KRONBORG, O., FENGER, C., OLSEN, J., BECH, O. & SONDERGAARD, O. (1989). Repeated screening for colorectal cancer with fecal occult blood test. A prospective randomized study at Funen, Denmark. *Scand. J. Gastroenterol.*, **24**, 599.

MACRAE, F.A., ST JOHN, D.J.B., CALIGIORE, P., TAYLOR, L.S. & LEGGE, J.W. (1982). Optimal dietary conditions for Hemoccult testing. *Gastoenterology*, **82**, 899.

MANDEL, J.S., BOND, J.H., SNOVER, D. & 5 others (1988). The University of Minnesota's Colon Cancer Control Study. In *Screening for Gastrointestinal Cancer*, Chamberlain, J. & Miller, A.B. (eds) p. 17. Hans Huber Publishers: Toronto.

ROTHMAN, K.J. (1986). *Modern Epidemiology*. p. 259. Little Brown & Co.: Boston.

SHIMIZU, S., TADA, M. & KAWAI, K. (1987). Evaluation of the utility of an immunochemical fecal occult blood test, OC-Hemodia-Eiken. *J. Kyoto Pref. Univ. Med.*, **96**, 1987.

ST JOHN, D.J.B., YOUNG, G.P., CUTHBERTSON, A.M. & 5 others (1989). Detection of colorectal neoplasia: comparison of guaiac, porphyrin, and immunochemical tests for occult blood. *Gastroenterology*, **96**, A492.